# Genetic variation in the brooding brittle-star: a global hybrid polyploid complex?

**DOI:** 10.1098/rsos.240428

**Published:** 2024-08-07

**Authors:** Andrew F. Hugall, Maria Byrne, Timothy D. O'Hara

**Affiliations:** ^1^ Museums Victoria, GPO Box 666, Melbourne, Victoria 3001, Australia; ^2^ School of Life and Environmental Science, University of Sydney, Camperdown, New South Wales 2050, Australia

**Keywords:** Ophiuroidea, allopolyploidy, marine, asexuality, sperm

## Abstract

The widespread and abundant brooding brittle-star (*Amphipholis squamata*) is a simultaneous hermaphrodite with a complex mitochondrial phylogeography of multiple divergent overlapping mtDNA lineages, high levels of inbreeding or clonality and unusual sperm morphology. We use exon-capture and transcriptome data to show that the nuclear genome comprises multiple (greater than 3) divergent (π > 6%) expressed components occurring across samples characterized by highly divergent (greater than 20%) mitochondrial lineages, and encompassing several other genera, including diploid dioecious species. We report a massive sperm genome size in *A. squamata*, an order of magnitude larger than that present in other brittle-stars, and consistent with our SNP-based measure of greatly elevated ploidy. Similarity of these genetic signatures to well-known animal systems suggests that *A. squamata* (and related taxa) is a hybrid polyploid asexual complex of variable subgenome origins, ploidy and reproductive mode. We discuss enigmatic aspects of *A. squamata* biology in this light. This putative allopolyploid complex would be the first to be reported from the phylum Echinodermata.

## 1. Introduction

Whole genome duplication (polyploidization) is a dramatic genetic rearrangement that is surprisingly well tolerated in some groups of eukaryotes [1]. One pathway to polyploidy is through hybridization (allopolyploidy). This is rare in animals and very often associated with asexual complexes [2,3], which often have much large population sizes and more extensive geographical ranges than their diploid sexual relatives [4,5]. In the aquatic environment, polyploidy has been noted in widespread asexually reproducing hermaphroditic bivalve species [6–9]. However, research is almost completely lacking in phyla such as echinoderms and cnidarians which are known to exhibit varied asexual reproductive strategies [2]. The study of polyploidy in echinoderms has been hampered by practical problems in observing cytogenesis, including small chromosomes, tight clustering, low mitotic index and difficulties in obtaining meiotic preparations [10]. However, genetic methodologies open an alternative path for research into hybridization and polyploidy (e.g. [4,11–15]).

**Figure 1.**
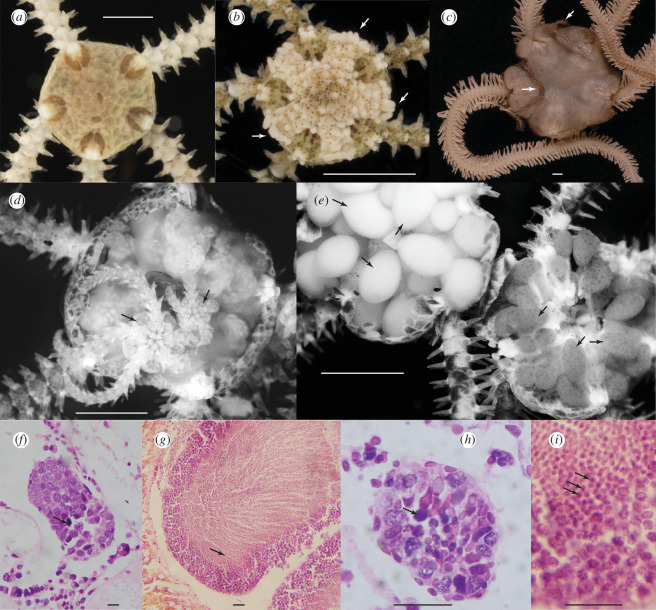
(*a*) *Amphipholis squamata*, dorsal disc and arm bases, (*b*) *Amphistigma minuta*, arrow indicates examples of the tubercle-shaped plates on the disc margin, (*c*) *Ophiodaphne formata,* arrows indicate the arms of the male emerging from underneath the disc, (*d*) *A. squamata*, dorsal disc removed to reveal brooded juveniles, (*e*) *Amphipholis pugetana*, dorsal disc removed to reveal dioecious gonads of male (upper left) and female (lower right), (*f*) *A. squamata* entire testis with only a few mature sperm (arrow) at a time, (*g*) *A. pugetana* testis with abundant sperm (arrow) dominating the lumen and (*h–i*) enlarged *A. squamata and A. pugetana* testis. Scale bars (*a–e*) 1 mm, (*f–i*) 0.02 mm.

Here we investigate potential allopolyploidy in the common and easy-to-culture marine brittle-star *Amphipholis squamata* (Delle Chiaje) from target-enriched next-generation sequencing data. *Amphipholis squamata* (figure 1*a*) is one of the most widespread and abundant benthic marine invertebrates. It is a small species; typically the central disc is less than 2.5 mm in diameter and the five arms measure less than 15 mm in length, feeding on organic detritus and plankton, and living under stones and among algal turfs and sessile invertebrates [16]. It has a nearly cosmopolitan distribution in coastal habitats, absent only from polar and brackish environments. It has been also reported from upper bathyal habitats across Atlantic, Indian and Pacific Oceans down to below 1350 m depth, but not from abyssal habitats [16]. This species has been called a biogeographic ‘paradox’ as it has achieved this enormous geographic range without a larval dispersal stage [17]. Instead, it releases live young (figure 1*d*), generally an indicator of limited dispersal capabilities. Its success is due to its ability to raft across oceans in algal holdfasts or on other coastal debris [16].

The ability of *A. squamata* to successfully colonize remote locations is undoubtedly enhanced by its life history. It is a simultaneous hermaphrodite, with both male and female gametes released via gonoducts into bursal sacs at the base of each arm, where fertilization is suggested to occur and development proceeds [18,19]. Individuals can reproduce in isolation [19,20]. Genetic evidence suggests a very high rate of self-fertilization or clonal development [21] which allows colonization of new localities from a single individual. The appearance of the sperm is highly unusual with the flagellum placed at a 70° angle to the spermatozoan axis which causes the sperm to swim eccentrically in three-dimensional spirals while slowly rotating about its own axis [19]. Fertilization has not been observed due to the sporadic release of single mature eggs [19]. The embryos develop from minute eggs into vestigial pluteus larvae that remain attached to the bursal wall where they are provided with maternal nutrients via the haemal sinus [22,23]. They metamorphose and grow into juveniles (figure 1*d*) that can reach a large size as they continue to be provisioned by the parent. They eventually leave the adult through the bursal slit [18]. One egg at a time is deposited in a bursa, embryos and juveniles of various sizes can co-occur in an adult [18], but release can be seasonal in many temperate populations [24]. Adults live for 1–2.5 years in the wild [24]. Brooding can facilitate rapid local increase in abundance, and *A. squamata* can reach densities exceeding 2000 animals per litre of algal matter in sheltered coastal lagoons [25].

Mitochondrial DNA from *A. squamata* forms a series of highly divergent widespread sympatric clades indicative of an ancient species-complex [17,26–29]. *COI* (K2P distance) divergence has been reported to exceed 23% [30], and so most mitochondrial DNA studies have focused on the slower evolving *16S* gene, where seven major lineages (mito-groups) (A–G) have been categorized [29,31]. Two of these groups (A and B) were found to be congruent with limited nuclear intron/microsatellite data and considered to represent biological species [28,29], although they were not consistent (except locally) with variable phenotypic characters based on skeletal shape, colour or bioluminescence [27,31,32]. Instead, many of these mito-groups are very widespread. Group A has been recorded from numerous temperate sites from the northeast Atlantic, Mediterranean, both coasts of the USA, South Africa, New Zealand and Chile [31]. B has a similar range, although it is encountered less frequently [31,33], C and D have only been reported from southern Australia and New Zealand, E (and possibly G) is circum-tropical, and F is only from Chile [31]. This diversity may be an underestimate of what is encompassed by this complex. Although the name *Amphipholis squamata* includes up to 30 synonyms [34], there are other similar morphological variants, including *Amphipholis sobrina* from Japan, and *Amphistigma minuta* from Southern Australia.

As part of a large exon-capture phylogenomic study of the Ophiuroidea [35,36], we sequenced a number of samples within and sister to the *A. squamata* complex. Several of these samples contained a surprisingly high rate of allelic heterogeneity [35]. Here we re-analyse those data to better reveal the underlying sequence variation. Combined with published information on *A. squamata*, we find that the genetic patterns are consistent with those described in well-known animal hybrid polyploid parthenogenetic complexes [9,11–14,37], i.e. high levels of apparent heterozygosity, evidence of clonality, many exons with more than two sequence variants, large phylogenetic divergence between sequence variants in the same individual that are incongruently distributed with respect to mitochondrial lineages, and polymorphism read frequencies that do not conform to diploid expectations. This would make *A. squamata* the first natural polyploid to be reported within the phylum Echinodermata.

## 2. Material and methods

The genetic data processing used here was derived from our ophiuroid exon-capture system [35,36,38]. Briefly, assembled transcriptome data were used to design a set of 120 bp probes tiled to target 1496 exons in 416 genes from DNA samples sequenced using an in-solution RNA target (exon-capture) enrichment procedure and Illumina 125 and 150 bp paired-end sequencing. Raw reads were de-duplicated (Clumpify [39]), trimmed (Trimmomatic [40]) and mapped (BLAT [41]) against a special composite de novo assembled (Trinity [42] or Tadpole [39]) sample-specific reference (see electronic supplementary material, table S1 for results and [35] for details on methods).

Our phylogenomic dataset, as of writing, includes 50 transcriptomes and 1946 exon-capture samples. For this study, we have included one transcriptome and 24 exon-capture samples focused around *A. squamata* (Order Amphilepidida, Family Amphiuridae) and close relatives (table 1, figure 1, electronic supplementary material, table S1). The tissue samples were sourced from ethanol-preserved museum specimens. Brooded juveniles and gonads were removed where observed.

**Table 1.** Description of samples and summary of sequence variant analyses. seq vars = sequence variants, NML = non-monophyletic lineages (see figure 2 caption for examples).

					single read (110 bp) analyses				merged read (190 bp) analyses			
genus	species	sample	locality	mito-group	no. exons	read coverage	median no. seq vars	proportion of exons with >2 seq vars	no. exons	median nucleotide diversity	median maximum divergence	median NMLs
*Amphipholis*	*squamata*	F211339	Australia, Victoria, Anglesea, 0–1 m	A	360	221	4	0.82	197	0.048	0.068	3
*Amphipholis*	*squamata*	F254622	Australia, Victoria, Gabo Island, 0–1 m	A	358	1359	4	0.82	200	0.046	0.068	3
*Amphipholis*	*squamata*	MRG808.F222771	Australia, Victoria, Port Welshpool, 0–1 m	A	350	102	4	0.81	15	0.040	0.053	3
*Amphistigma*	*minuta*	F173962	Australia, Victoria, Flinders, 0–1 m	A	304	70	3	0.72	104	0.052	0.075	2
*Amphistigma*	*minuta*	F211345.2	Australia, Victoria, Flinders, 0–1 m	A	358	163	3	0.77	183	0.061	0.088	2
*Amphipholis*	*squamata*	GLB.009	Australia, Victoria, Lakes Entrance, 1–2 m	A	158	56	3	0.75	22	0.029	0.039	3
*Amphipholis*	*squamata*	A65682	South Africa, Moullie Point, 0–2 m	A	314	69	3	0.72	97	0.033	0.046	3
*Amphipholis*	*squamata*	DZMB53422C	South of Iceland, 1588 m	B	282	48	3	0.58	79	0.028	0.039	2
*Amphipholis*	*squamata*	DZMB55448B	South of Iceland, 214 m	B	359	373	3	0.65	197	0.034	0.045	3
*Amphipholis*	*squamata*	IE.2013.10780	New Caledonia, Ile des Pins, 493–694 m	C	359	679	1	0.03	199	0.012	0.012	1
*Amphipholis*	*squamata*	Misaki029	Japan, Sagami Bay, 109–160 m	C	360	743	3	0.5	200	0.027	0.037	2
*Amphipholis*	*sobrina*	Sagami39	Japan, Sagami Bay, 218–318 m	C	348	361	2	0.42	164	0.021	0.023	1
*Amphipholis*	*squamata*	Misaki011	Japan, Misaki, 0–1 m	E	358	1204	3	0.68	200	0.029	0.042	2
*Amphipholis*	*squamata*	MVF214040	USA, Florida, Blue Heron Bridge, 1–5 m	E	89	148	3	0.76	26	0.025	0.036	2
*Ophiodaphne*	*formata*	F111999	Australia, Ningaloo, 165–166 m	S	345	102	1	0.04	172	0	0	1
*Ophiodaphne*	*materna*	IE.2007.3135	New Caledonia, Munida Seamount, 233 m	S	353	119	1	0.02	150	0	0	1
*Ophiodaphne*	*scripta*	IE.2007.5337	New Caledonia, Capel Bank, 285–545 m	S	349	113	1	0.02	178	0	0	1
*Ophiosphaera*	*insignis*	IE.2007.7571	New Caledonia, Chesterfield Plateau, 345–377 m	S	332	85	1	0.02	155	0	0	1
*Amphipholis*	*linopneusti*	IE.2007.7480	New Caledonia, Chesterfield Plateau, 519–522 m	S	206	56	1	0.02	86	0	0	1
*Amphipholis*	sp2	AM J24929.2	New Zealand, Raoul Is, 21 m	S	177	57	2	0.16	87	0.013	0.013	1
*Amphipholis*	*pugetana*	SIO E7449	Unites States, California, Redondo Knoll, 575 m	X	352	208	1	0.02	191	0.011	0.011	1
*Amphipholis*	*pugetana*	SIO E7946−2	Unites States, California, Hancock Bank, 455 m	X	354	665	1	0.02	195	0	0	1
*Amphipholis*	*torelli*	DZMB49260B	Faroe Island Ridge, 400 m	X	340	871	1	0.01	185	0	0	1
*Amphioplus*	*depressus*	KGR23021	Australia, W Australia, King George River, 37 m	X	296	153	1	0.01	137	0	0	1
*Amphipholis*	*januarii*	UF11904	French Antilles, St. Martin, Caye Verte, 1–9 m	X	186	36	1	0.01	41	0	0	1

Our probe target also included 1431 bp of the mitochondrial *COI* gene. However, as mitochondrial *16S* (rather than *COI*) has been used previously to categorize *Amphipholis* lineages, we also assembled partial fragments of this gene from a transcriptome (sample MVF214040), Sanger sequencing using universal primers 16Sar and 16sbr (F211339, F173962, GLB009, F211345-2, F222771, AMJ24929) and the remaining from off-target reads of the exon-capture samples (electronic supplementary material, table S1).

### 2.1. Assessing within genome diversity

To avoid the complications of attempting to assemble highly heterozygous genomes from short reads, we assessed allelic diversity directly from the Illumina paired-end reads as sequence variants, using a custom pipeline (RHACK3, see electronic supplementary material) developed from our exon-capture system [35]. Reads were pre-processed (as above) and mapped using BLAT [41] software with relaxed settings (allowing matching of up to 10% sequence difference) against a composite sample-specific reference (Misaki011, IE.2013.10780 and DZMB49260B, table 1).

We ran two series of analyses using (i) single reads and (ii) merged reads formed from pairs where left and right reads sufficiently overlapped (using FLASH software [43]). The first dataset was used for analyses demonstrating sequence variant richness and divergence, and the second to achieve greater resolution in certain phylogenetic analyses. To determine sequence variants, we defined a fixed consistent window of 110 bp (single reads) and 190 bp (merged reads) near the centre of the exons (where coverage is typically greatest). Only reads that filled greater than 80% of this window were counted, and only exons with total coverage greater than 40 per sample were considered (greater than 30 for merged reads). Reads were then grouped into bins that allowed one base difference (for read error) with the most abundant base per site chosen to represent the bin consensus. This process provided aligned datasets of all sequence variants of all samples per exon. Out of our 1496 exons, there were 1255 exons of sufficient length to encompass a 110 bp window and 411 exons for a 190 bp window. This was reduced to 360 and 200 exons respectively (in 194/128 genes) that occurred in at least half the samples, including at least one of each mito-group (table 1). Average pairwise p-distance (nucleotide diversity, *π*) and median maximum p-distance were used to measure sequence variant diversity.

**Figure 2.**
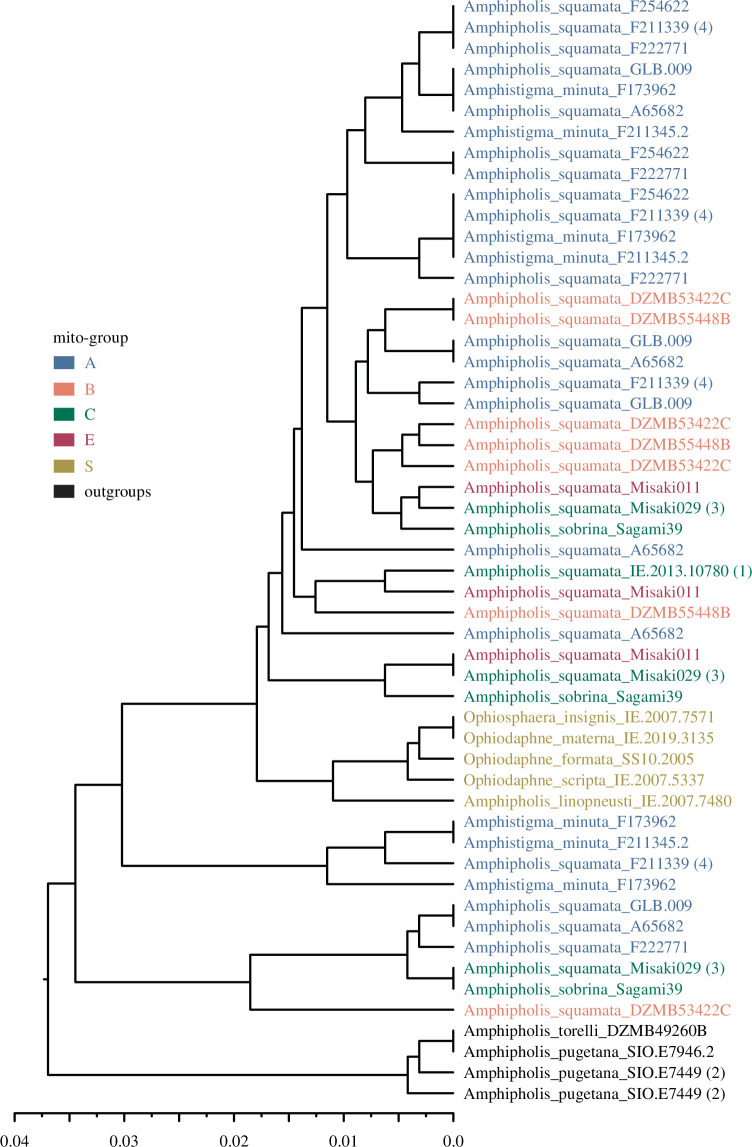
Example of an exon sequence variant phylogeny. UPGMA p-distance tree of a merged-read exon (ZGC73290 exon 1) colour-coded by highly divergent mito-group (figure 3). Note some samples missing due to inadequate coverage. Sample labels with numbers (1–4) indicate examples with that number of sequence variants. Putative diploid samples have one (homozygous, e.g. IE.2013.10780 and the *Ophiosphaera*-complex) or two (heterozygous, SIO.E7449) sequence variants. Putative allopolyploid examples have three (Misaki029) or four (F211339) sequence variants, some of which are non-monophyletic with respect to other mito-groups. We use the median number of these non-monophyletic lineages (NMLs) across exons to quantify hybridization within each sample (table 1). In this example exon, both Misaki029 and F211339 have an NML of 3, as two of the four variants in F211339 fall into a clade comprising only one mito-group (A, at top of tree). Note some sequence variants are identical across samples in different mito-groups, e.g. between Misaki029 (C) and Misaki011 (E).

The longer merged-read datasets were used to generate unweighted pair-group mean average (UPGMA) p-distance dendrograms (using PHYLIP v. 3.695 [44]) as estimates of sequence variant phylogeny (the limited number of sites not warranting a more complex method). UPGMA were used to (i) allow simple division into lineages based on a proportion of tree height, and (ii) rather than impose an *a priori* outgroup root, use the implied root to determine consistency with expected most distant nominal diploid outgroup. First, we used these trees to demonstrate the diversity of parental lineages possibly contributing to a hybrid genomic complex [45] (excluding a few exons where the ingroup and outgroup samples were not reciprocally monophyletic). We then calculated the number of non-monophyletic clades of each mito-group that were formed from sequence variants for each sample in each exon tree, i.e. the number of lineages that were separated by sequence variants from other mito-groups. For example, the three sequence variants from Misaki029 in exon ZGC73290 (figure 2) are all separated in the phylogeny by sequences from other mito-groups and so form three lineages that are non-monophyletic. We then calculated the median number of these lineages for each sample across all exons, which we term the number of non-monophyletic lineages (NMLs, table 1). These UPGMA trees were also used to calculate the minimum p-distance between sequence variants belonging to different mito-groups, including variants shared across groups (p-distance = 0), as additional measures of potential hybridization between the highly distinct major mitochondrial lineage groups.

To investigate ploidy more directly, we also generated a minor state frequency (MSF) distribution (profile) [13] for each sample, calculated as the ratio of read coverage of the second most common base at a site divided by the total read coverage, for all multi-state sites across the 1496 exons with coverage greater than 40 and less than 95th percentile of coverage across all sites (to exclude potential high copy number pseudogene artefacts). The modal shape of the MSF spectrum gives some inference on the copy number of subgenomes, despite some data noise from vagaries of gene expression, exon-capture efficiency and permissive base mapping [13]. A modal peak on the MSF spectrum near 50% is consistent with expectations of diploid, a peak at 33% with a triploid and so on [13].

### 2.2. Summary phylogenetic trees

We generated maximum likelihood phylogenetic trees for various subsets of mitochondrial and nuclear gene data, using IQTree v. 2.2.0 software [46] with ModelFinder selected optimal model and ultrafast bootstrap support (*n* = 1000). Mitochondrial data comprised (i) *16S* (combined GenBank accessions and our samples) aligned with MAFFT [47], and (ii) combined *16S* and *COI* for our samples only (1431 sites *COI*, 1314 sites *16S*). Nuclear data comprised concatenated consensus (IUPAC coded) sequences, filtered by the level of polymorphic sites and data incompleteness to 1325 exons in 416 genes (252.2 kb), and run with a codon position optimal model. This exon data was also used to generate 234 separate gene trees (using a simple TN93 model) then summarized in ASTRAL II (v. 5.5.10) [48] with local posterior support values [49].

To provide another way of summarizing overall genomic similarity between samples, we used the sequence variant UPGMA trees to define a matrix of the presence/absence of sequence variant lineages. For each exon, a set of lineages were defined by 1/3 of the total tree height, and then the occurrence of each of these scored (0/1) for each sample. These presence/absence matrices were then used to generate neighbour-joining (NJ) dendrograms and non-metric multi-dimensional scaling (nMDS) plots (using metaMDS() in the R library vegan [50]). For this, we used the best sampled 70 exons (to minimize missing data).

### 2.3. Flow cytometric estimation of genome size

Five ophiuroid species *Amphipholis squamata* (not sequenced, but presumably clade A or E based on its location), *Ophionereis schayeri* (Müller and Troschel), *Macrophiothrix spongicola* (Stimpson), *Clarkcoma pulchra* (Clark) and *Ophiactis resiliens* Lyman and one sea urchin *Heliocidaris tuberculata* (Lamarck) were collected from around Sydney in shallow water (1–2 m depth). The testes of *Amphipholis* were detached from their attachment to the genital plates. As the testes were small (approx. 100 µm diameter) and sperm were not evident, they were checked microscopically for the presence of sperm. The sperm were teased out of the testes using microneedles and micropipettes. Each testis has only approximately 1000 sperm and so this process was repeated with 30 isolated testes dissected from 20 *Amphipholis* specimens. These samples were pooled through necessity to isolate sufficient sperm for analysis. We could not obtain sufficient numbers of somatic cells from *A. squamata* for comparison. The other ophiuroid species had large testes filled with sperm which oozed out. The sperm of these species were collected using a glass pipette placed in a tube and kept dry at 4°C until used for flow cytometry. The sperm of *H. tuberculata* was collected from the aboral surface of the test following injection of 0.5 M KCl, and stored dry until use.

The absolute quantity of DNA per cell in picograms was estimated for the sperm of the five ophiuroid species by flow cytometry with an ICP 22A (Ortho Instruments). Sperm solutions were prepared by adding a few microlitres of dry sperm to 5 ml of 0.9% tri-sodium citrate. The fluorescence intensity in picograms of DNA per channel was calibrated using two standards of known DNA content, with *Drosophila melanogaster* diploid cells used as the lower genome size standard (0.36 pg DNA) [51] and the sperm of *Heliocidaris* as upper size standard (1.05 pg DNA) [52]. A drop of each standard was added to 2 ml of staining solution containing 1% Triton X-100 and 1 µg ml^−1^ of 4',6'-diamidino-2phenylindoel 2 HCl (DAPI) (Mannheim Boehringrer) in 0.08 M phosphate buffer pH 7.3 and used within 1 h (being stable for over 2 h). Drops of the *Drosophila* standard*, Heliocidaris* standard and ophiuroid sperm were added sequentially to the cytometer for each run.

### 2.4. Testis histology

For histological examination of the testes, *Amphipholis squamata* and *A. pugetana* were collected near Bamfield, Vancouver Island, British Columbia, Canada. *Amphipholis squamata* were also collected from Belize. Whole discs of *A. squamata* and dissected testes of *A. pugetana* were placed in Bouin’s fixative and processed for wax histology. The blocks were sectioned (6–7 µm thick) and the sections were stained with haematoxylin and eosin. The sectioned testes were examined with an Olympus microscope, photographed with an attached camera, and sperm nucleus size was measured.

## 3. Results

### 3.1. Genetic data quality

Exon coverage varied substantially (table 1) but most samples had more than sufficient sequence variant scoring (cf. [53]), with some expected reduction when merging reads (proportion merged varied between 13% and 88%). To minimize missing data, we used various combinations of samples and loci in different analyses, an inevitable compromise with such heterogeneous data, and relied on the consistency of major patterns to reinforce our main conclusions. Due to the inherent bias in gene expression, the transcriptome sample (*Amphipholis_squamata*_MVF214040) had fewer recovered exons, and a different mix, dominated by ribosomal protein genes. Our samples appear to contain very low levels of contamination judging from the near-unanimous read coverage for a single *COI* haplotype in all samples (greater than 1000-fold read-coverage ratio).

**Figure 3.**
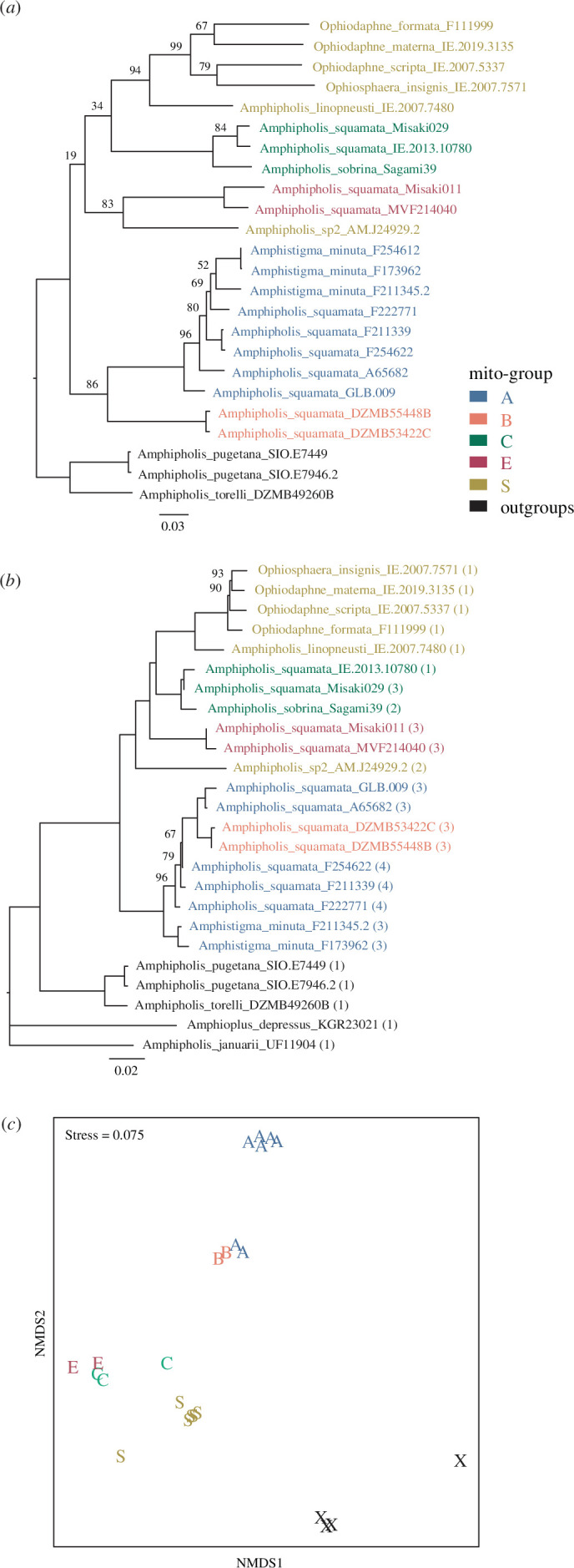
Summary phylogenetic patterns: (*a*) mitochondrial DNA (*16S* + *COI*) representing the maternal subgenome, (*b*) standard concatenated 252 kb IUPAC-coded exon data—with numbers in brackets indicating median number of sequence variants across exons (see figure 4), (*c*) shared sequence variant cluster lineage Jaccard pairwise distance from 70 data-rich exons as nMDS, X = outgroups. Panels (*b–c*) provide alternative ways of representing the amalgam of parental subgenomes. Samples are colour-coded according to *squamata* complex mito-group classification. Trees (*a*) and (*b*) are IQ-Tree ML with bootstrap node support. Some samples are missing from some trees due to data limitations. The ASTRAL-II tree version of (*b*) is essentially the same (electronic supplementary material, figure S2).

In the initial standard exon-capture mapping (electronic supplementary material, table S1), almost all samples named as *Amphipholis squamata*, *A. sobrina* and *Amphistigma minuta* showed anomalously high levels of polymorphic sites compared with outgroups and related dioecious (e.g. *Ophiodaphne*) species. As described below, we subsequently re-analysed the exon capture data to better reveal the underlying sequence variation. Despite limited sampling, we confirm previous reports of very high divergence of the major lineages, with *COI* differences exceeding 20% (and 7% within mito-groups), matched by maximum nuclear exon differences exceeding 8% (figure 3*a,b*).

### 3.2. Mitochondrial clade identity

We assigned a mitochondrial clade identity to each of our samples through a tree-based comparison of our *16S* sequence data with published data [17,26–28,31] (electronic supplementary material, figure S1). For the purposes of analysis, we designated six mitochondrial (mito-) groups as a means of classifying samples that share maternal genomic lineages: previously identified groups A, B, C and E, related taxa (S) and outgroups (X). *16S* sequences in animals we identified as *Amphistigma minuta* clustered within group A. Similarity of *COI* places *Amphipholis sobrina* Sagami39 within the group C (figure 3*a*).

Our samples were generally found within known geographical and bathymetric ranges of the *16S* groups. Our B samples came from mid-upper bathyal sites south of Iceland and are distinct enough to be recognized as their own subgroup (B2). The B sample from 1588 m is one of the deepest yet recorded for the whole *A. squamata* complex.

We considerably extended the known range of group C, to include outer shelf and upper bathyal (109–694 m) sites in New Caledonia and Japan. One of the C samples was identified as *A. sobrina* and our other samples of C also had this morphology (a tendency to have four arm spines, enlarged radial shields, distinct primary disc plates and thinner arms compared with the typical *A. squamata*). *Amphipholis sobrina* has previously been reported from Japan and the China Sea (20–550 m) [54] and group C from 10 to 100 m off southern New Zealand [27]. In summary, *A. squamata* complex differs from previously reported marine polyploid examples in its extensive depth distribution (0–1600 m). Depth-based genetic divergence and speciation is common among marine lineages [55], and here we document distinct bathymetric ranges for some of the mito-groups.

### 3.3. Phylogenetic analyses

While the mtDNA tree provides an estimate of the maternal lineage component of the whole genome, in hybrids the nuclear data can be a complex amalgam of subgenomes not easily resolved as a phylogenetic network [56]. Hence we provide only what are in effect summaries of overall nuclear genome exon similarity.

In all phylogenetic trees based on nuclear exon data (figure 3*b*, electronic supplementary material, figure S2) we recovered two main groups, or clades, within the *A. squamata* complex: the first containing A, B and *Amphistigma minuta* and the second containing C (and *A. sobrina*), E, an undescribed species (sp2) from the Kermadec Islands, and a group of sexually dimorphic species (often with dwarf commensal males, figure 1*c*) that are epizoic on sea urchins including *Ophiodaphne* and *Ophiosphaera* species and *Amphipholis linopneustei*, which we will refer to as the *Ophiosphaera* complex (as the earliest genus name). Excepting within the A–B group, the major lineages generally have high bootstrap support. Allowing for high (saturated) divergence and limited support, the mtDNA tree is broadly similar (figure 3*a*, and electronic supplementary material, figure S2) but with a key difference in the relative divergence between A and B and between C and E. While support for relationships among the major mtDNA lineages is weak, the lineages themselves are highly distinct and thus must represent substantially diverged maternal nuclear genomes at some point in the history of the complex.

*Amphistigma minuta* was generally embedded in the A–B clade and was only sister to this group in the exon data (concatenated exons, figure 3*b*, electronic supplementary material, figure S2). This was a surprise as *A. minuta* specimens are easily distinguished morphologically by the presence of elevated plates around the rim of the disc (figure 1). The A and B mito-groups were only reciprocally monophyletic in the mtDNA tree (figure 3*a*, electronic supplementary material, figure S1). Within the C–E main clade, sp2, C (= *sobrina*), E and the *Ophiosphaera* complex formed distinct subclades, although their relationship differed between analyses.

*Amphipholis pugetana* and *A. torelli* formed a clade that was sister to the *A. squamata* complex. *Amphioplus depressus* and *Amphipholis januarii* were much more distant outgroups, not closely related to the *A. squamata* complex. The genus *Amphipholis* has been shown previously to be polyphyletic with respect to other amphiurid genera [36] and a genus-level taxonomic revision is required.

### 3.4. Allelic richness and diversity

**Figure 4.**
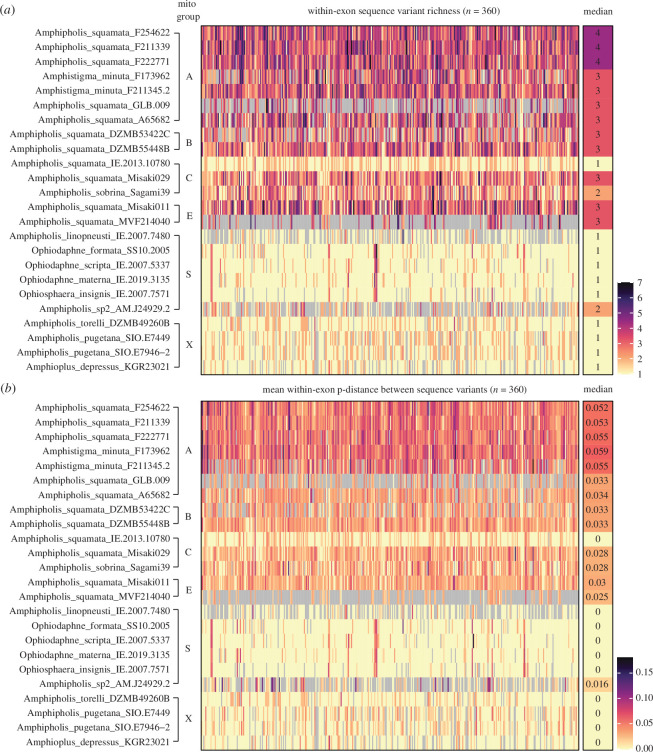
Heat-map of sample sequence variant (*a*) richnessand (*b*) mean within-exon pairwise p-distance (nucleotide diversity *π*) between sequence variants from our 360-single-read exon dataset. Last column is the median across exons for each sample. Note the consistency between exons within a sample and between samples within mito-groups (including group E that contains a transcriptome MVF214040).

The proportion of loci with more than two sequence variants was very high for most samples in the *A. squamata* complex (figure 4*a*, table 1, greater than or equal to 60%) which implies whole genome duplications or hybrid polyploidy. The median number of variants per exon per sample ranged from 1 to 4 (table 1, figure 4*a*, electronic supplementary material, figure S3), with the highest numbers (3–4, with up to 7 variants for some exons) occurring in the A mito-group. Samples from groups B and E had slightly fewer variants (median 2–3), and C and sp2 fewer again (1–3). There was considerable variation within our group C samples, with sample Misaki029 having a median of 3, Sagami39 with 2 and IE.2013.10780 with only 1. Finally, the majority of exons in the *Ophiosphaera* complex and outgroups were homozygous, with only a few having more than three variants. The different sequent variant datasets were highly consistent (electronic supplementary material, figure S4).

This pattern of variant richness was also reflected in variant nucleotide diversity (table 1, figure 4*b*, electronic supplementary material, figure S3), which ranged up to (an exceptional) 6% in the A group (including *A. minuta*), and 4% in C and E. Median maximum divergence across groups A–E can exceed 8%. The transcriptome (MVF214040, mito-group E) sample showed sequence variant diversity consistent with that obtained from our exon-capture E sample (Misaki011), hence these variants were expressed (transcribed as RNA), discounting bias from junk DNA pseudogenes. Again, sample IE.2013.10780 had low diversity. *Amphipholis* sp2 showed a mixed signal of elevated richness and nucleotide diversity despite some missing data. Diversity was typically low (less than 1%) in the *Ophiosphaera* complex and outgroups.

To provide further inference on possible hybridization, we assessed the evidence for combinations of different lineages, using each sample’s mito-group as a maternal marker. Variants within a sample appear to be related to more than one mito-group (e.g. figure 2). There was a median of 2–3 NMLs within samples in the A–B–*A. minuta* group and 1–2 in the C–E group (table 1, electronic supplementary material, figure S5). The short (150–190 base) exon reads have limited ability to resolve putative subgenomes, but the divergence scale is quite high (approx. 8%; median UPGMA tree height 5%) among the major lineages within a sample (and as demarked by the major mito-groups). Thus, we specifically focus on two classes of variants within an individual that (i) are divergent (greater than 5%) and separated by variants from different mito-groups, or (ii) are very similar (less than 1%) to variants from different mito-groups. While some exons may fail to correctly resolve due to incomplete lineage sorting or stochastic mutation effects, across the whole set of exons the median result should be reasonably robust to such idiosyncratic artefacts. The majority of exons show the contrasting patterns of high (i) and low divergence (ii) for samples of mito-groups A and B. Similarly for C–E. However, few (less than 10%) exons show closely related variants between these two pairs. Across all exons, the median minimum distance between A–B and C–E variants is 0.006 and 0.013 but between any of the two pairs is 0.03 (electronic supplementary material, figure S6).

Cluster and ordination of the presence–absence of sequence variants in each sample differed from the mtDNA maternal lineage phylogeny for some A and B mito-group samples (figure 3*c*). For example, samples GLB.009 and A65682 appear to be more similar to B samples than other A samples (despite the large difference in mtDNA).

### 3.5. Minor state frequency profiles

**Figure 5.**
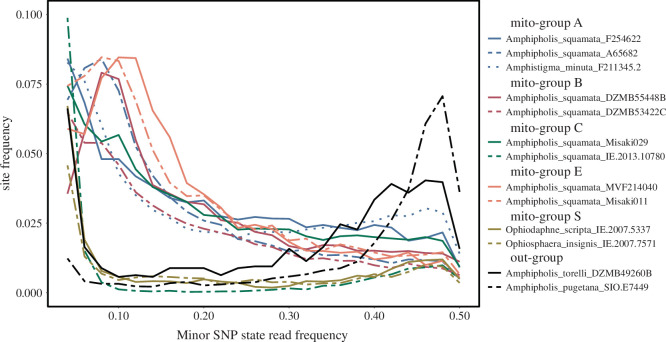
Minor state frequency (MSF) distribution of SNPs plotted in 0.02 (2%) bins for selected exon-capture and transcriptome (MVF214040) samples. Minor state is calculated as the ratio of coverage of the second most common base at a site divided by the most common, for all sites with more than one base state recorded. All sites with coverage greater than 40th and less than 95th percentile. SNP frequencies less than 0.04 are not shown. MSF distributions with a peak around 0.5 (50%) conform to diploid expectations (group S, outgroups and the group C sample IE.2013.10780), other samples have MSF distributions indicative of elevated ploidy [13].

The *Ophiosphaera* complex, our outgroup samples and *A. squamata* sample IE.2007.10780 (mito-group C) showed peaks approaching 50% on MSF profiles (figure 5) consistent with diploidy. In contrast, other samples from groups A, B, C and E all had peaks at less than or equal to 12.5%, or a broad upward trend towards low values, consistent with ploidy levels far exceeding four or being complex aneuploid genomes. The variation in these profiles suggests potential differences in ploidy between samples. *Amphipholis squamata* IE.2007.10780 may represent a sexual parental lineage.

### 3.6. Size of genome and sperm nucleus

The sperm of *A. squamata* had a genome size of 27.18 pg, approximately an order of magnitude larger than the sperm of the other species tested, *Clarkcoma pulchra* (3.51 pg), *Ophionereis schayeri* (3.13), *Macrophiothrix spongicola* (1.80) and *Ophiactis resiliens* (2.81). These four comparison species possessed large testes and are known to have a broadcast spawning mode of reproduction [57,58]. The sperm nucleus of *A. squamata* had a mean diameter of 5.5 µm (s.e. = 0.18, *n* = 9), while the sperm nucleus of *A. pugetana* had a mean diameter of 2.6 µm (s.e. = 0.08, *n* = 9). Measurement of the sperm nucleus in published transmission electron microscopy sections of *A. squamata* [19] also showed that they are large, 6.7 µm in diameter.

## 4. Discussion

While our data has limited power and was never intended to explicitly resolve subgenome hybrid polyploidy (e.g. [56]), in the appropriate context it strongly implies that *A. squamata* encompasses a hybrid polyploid complex. This context includes comparison of genetic patterns to other better-known hybrid polyploid complexes, and also previous work on *A. squamata* itself. Considering the unknown complexity and likely under-sampling of the system, we discuss broad inference of hybrid origins from the dominant patterns of sequence variants within and between mito-groups. While this is not an explicit phylogenetic network, it is a robust conservative inference that there must have been hybridization, and probably multiple times.

The presence of numerous sequence variants (greater than 2) in a high proportion of our target loci (figure 4*a*) is indicative of extensive genomic duplication or polyploidy occurring in the *Amphipholis squamata* complex. Both the richness of exonic sequence variants (median at least 3, up to 7) and MSF minor-state frequency profiles [13] imply ploidy levels of more than 4. The massive genome and nucleus size recorded from the sperm of *A. squamata* compared with other species is also consistent with high ploidy, although, by itself, this could also indicate an excess of non-coding regions. Sequence variant richness is reduced in other *A. squamata* samples (median ranging from 4 to 1) and one (IE.2013.10780) appears to be diploid. There is no evidence for polyploidy in the *Ophiosphaera* clade, *Amphipholis* sp2 and outgroups. Thus, the data suggests variation in the number of genome duplication events across the complex. The presence of highly divergent nuclear sequence variants (greater than 8% p-distance) is indicative of hybridization between divergent lineages (allopolyploidy) and/or ancestral genome duplication. These values are among the highest yet recorded [14] (once bdelliod rotifers are discounted [59]).

In the individuals with apparent high genetic diversity, it is worth considering how much variation is ancestral and how much owing to distinct hybridizations. We suggest that a single ancestral event cannot explain all of the patterns we see because (i) inference from having more than two distinct variants related to more than one mito-group in many samples (table 1 median NML 3); (ii) that the pattern of both divergent lineages and identical sequences in different mito-groups within a sample is not consistent with them all being inherited at the same time; (iii) that these comprise at least two major groups: the AB and the CE mito-groups; and (iv) the presence of diploid taxa between these two groups, and in the case of the C, even within the group (sample IE.2013.10780). This implies at least two independent origins (the AB and CE polyploids) and probably at least two events within each (to get NML greater than 2 and to account for the diploid sample C). All of this is consistent with well-studied animal allopolyploids, of multiple complex origins, along with closely related sexual diploid lineages [1,2,4,11–14,37,45,60]. Our supposition is that, with further sampling, sexual diploids may be found across the *A. squamata* complex, similar to mito-group C.

Genetic uniformity among *A. squamata* offspring has been reported several times as reflecting selfing or clonality. Poulin *et al*. [61] used multi-band ‘fingerprint’ random amplified polymorphic DNAs (RAPDs). Boissin *et al*. [21] observed that several loci (four of seven) had more than two bands but excluded these loci in their analysis of heterozygosity in one lot of individuals. They did a second analysis with another lot of individuals using all seven loci together based on similarity of a combined ‘fingerprint’ pattern. Thus, both these studies were necessarily blind to polyploidy, and not inconsistent with our results.

The presence of multiple and variable genome duplications and hybrid events are indicative of allopolyploid cascades. In animals such as *Aspidoscelis* (whiptail) lizards and *Ambystoma* salamanders, hybrid polyploids can remain fertile and backcross with diploid progenitors, resulting in offspring with increasingly elevated ploidy levels (until they eventually become sterile) [37]. Such complexes contain a mix of lineages of varying fertility, ploidy and subgenome ancestry, which can be quite cryptic without detailed diagnostic analysis. Thus, the previous genetic studies that indicate that *A. squamata* is clonal or self-fertilizing only apply to those individuals sequenced. Among our small sample set, we did find one individual that appears to be diploid. Some diploid ancestors may no longer exist, or, as in other complexes, fully sexual diploids may occur at a low frequency and go unnoticed without detailed genetic analysis [1,4,5,60], particularly for hermaphrodites with male gametes that may not be fully functional.

We have no direct evidence for the age of *A. squamata* hybridization or ploidy-elevation events. The sharing of almost identical (less than or equal to 1 bp variation) sequence variants between mito-groups A and B, and C and E, suggest relatively recent hybridization with little subsequent divergence from mutation. However, we also have very high mitochondrial and nuclear sequence diversity (exceeding 20% K2P distance in mitochondria and greater than 10% across our nuclear target) indicating considerable age—in the millions of years—of the component lineages (or species) contributing to the complex, as has also been reported for several other groups [11,14,60]. There are no reported fossils for this complex, but node age estimates from published phylogenies are Palaeogene in age [26,36].

The varying topologies and measures of similarity produced from different sequence datasets and methodologies (figure 3, electronic supplementary material, figure S2) may be indicative of varying combinations and proportions of maternal and paternal ancestral subgenomes contained in our samples. Samples that are divergent on the mtDNA phylogeny can be brought together in our summary measures of nuclear data. For example, the group A samples A65682 and GLB.009 are closer to B group samples in the nuclear than in the mitochondrial DNA analyses, reflecting that while they share the same maternal lineage (A) these samples would have a greater proportion of B group paternal subgenomes. The distinctness of *A. minuta* and A group samples also varies between analyses, as does the relationship between C and E samples. These kinds of patterns are consistent with the combinations of subgenomes seen in better-studied hybrid polyploid taxa [37].

Buried in the *A. squamata* complex is a lineage of putatively diploid dioecious species (the *Ophiosphaera* complex) which (for ophiuroids) exhibit unusual sexual dimorphism and host (echinoid) associations. This lineage does not seem to have participated in the *A. squamata* hybridization events, but it is a fascinating addition to the evolutionary complexity of the system. The diploid sister taxa we have sequenced also seem unrelated to the polyploid swarm, although, remarkably, *A. torelli* is six-armed, asexually fissiparous, hermaphroditic and retains its larvae within the bursae until at least the blastula stage [62]. Its sister species from the west coast of America, *A. pugetana*, is sexually dioecious (figure 1*e*).

Allopolyploidy is often associated with asexual reproduction such as parthenogenesis [3]. However, we do not have conclusive evidence for this in *A. squamata*. While genetic studies have repeatedly confirmed that the vast majority of juveniles are identical to their mother [21,29] and that ‘virgin-births’ are possible [19,20], all studies of *A. squamata* have found it to be a simultaneous hermaphrodite with paired female and male gonads (but with aberrant sperm). However, it is not known if the sperm of *A. squamata* have a reduced genome compared with somatic cells. The large size of the sperm of *A. squamata* compared with that of its broadcasting congener *A. pugetana*, and its large DNA content, points to the possibility of non-reduced sperm, although processes that resemble meiosis have been observed in the testes of *A. squamata* [19]. The large sperm nucleus may complicate meiosis [1]. The egg cells have also been described as having relatively large nuclei [18]. In corbiculid brooding bivalves with asexual clonal-hermaphroditic reproduction the sperm are non-reduced and have the same DNA content as the somatic cells [6,7].

Reproductive assurance [2] is also possible through selfing or sperm-dependent reproduction. Observed *A. squamata* sperm have an unusual flagella that inserts into the sperm head at an oblique angle and so are not fully motile [19]. This may be a feature that promotes self-fertilization [19]. Nevertheless, we have presented evidence for hybridization in this article, indicating that outcrossing must have been successful in some circumstances. Another possibility is *A. squamata* has sperm-dependent asexual reproduction such as gynogenesis or androgenesis, where sperm is required for embryogenesis but the egg and sperm do not undergo fertilization [63], as found for corbiculid bivalves. Asexuality may be facultative. Moritz & Bi [37] speculate that the formation of polyploid cascades through occasional backcrossing may be facilitated in lineages where sperm are still required for egg development.

The mechanism of allopolyploid formation varies considerably between taxa [1,2]. Polyploidy has been rarely reported from echinoderms, although historically this may be due to the difficulty of undertaking cytogenetic studies in this phylum [10,15]. On the other hand, asexuality does occur within echinoderms via splitting of organisms into two pieces—in adults of ophiuroids, asteroids and holothuroids [64] and in the larvae in all classes except crinoids [65]. Many of these species also show evidence of facultative sexual reproduction. Conversely, parthenogenesis in female-only echinoderm populations has been reported more rarely [66].

Increased ploidy, hermaphroditism, small body size, brooding and non-reductive sperm are noted for some bivalve species and is considered to contribute to their success [67]. *Lasaea* bivalves are a marine example of an allopolyploid swarm exhibiting variable polyploidy (3–6*n*), multiple hybridization events and parthenogenesis [8,9]. Most of its global range supports various sympatric parthenogenic/gynogenetic lineages with known planktotrophic or direct-developing diploid populations restricted geographically [68,69]. Although analyses of mtDNA have estimated that the various *Lasaea* lineages diverged in the Neogene [69], hybridization events may be more recent [9].

The *A. squamata* complex shares many ecological features with these and other allopolyploids. First, polyploid lineages often have a more extensive geographic range than their diploid progenitors [5]. *Amphipholis squamata* is an excellent colonizer and has achieved a widespread distribution despite not having a dispersive phase in its life cycle, possibly assisted through reproductive assurance. Its polyploids occur throughout the wide geographic range but it is unclear where the potential diploid lineages might be found. Several other aspects of *A. squamata* biology, such as abundance varying with environment [25], colour variation and predation [70], and parasite load [71], might usefully be re-evaluated in the light of our hypothesis, considering the extensive theory and knowledge in other systems [1,2,5,60,72,73].

## 5. Future directions

In summary, we have information that is entirely consistent with at least some components of the *A. squamata* complex containing some, or all, of these features: (i) polyploid, (ii) hybrid, (iii) clonal, (iv) giant genome, and (v) deviant male gametes. Thus *A. squamata* has the potential to become a useful organism to study the evolutionary and ecological advantages of polyploidy, hybridization and reproductive assurance in a global marine system. It is easy to collect, culture and propagate in aquaria. One of the main goals of this article is to promote future research into this fascinating complex.

Regarding the genetic data, what is needed is much better sampling of individuals and of subgenomes. We are fairly sure there are more major lineages to be accounted for, as indicated in the work of Sponer [31] and Boisson *et al*. [28,29]. We need a larger number and better geographic coverage of samples to better represent the genetic diversity of this complex and, in particular, potential diploid sexual elements. If other hybrid complexes are a guide, diploids will be at low abundance and/or have a restricted range [72]. Moreover, we do not have an adequate understanding of the range of the polyploid lineages, nor have we yet found any obvious diploid–hybrid intermediates. Finally, long-reads from third-generation sequencers (e.g. [59]) are required to generate long haplotypes with the power to better resolve subgenome divergence and relationships, and to have any chance of teasing apart ancestral and derived variation owing to post-hybridization mutation (the Meselson effect), partial gene conversion, recombination or inherited genetic diversity from parental lineages [11,13,14,74].

We are currently unsure if individuals of *A. squamata* self-fertilize, are parthenogenetic or use some sort of sperm-mediated strategy. This understanding has been hindered historically by the difficulty of obtaining ripe eggs [18] and useful cytogenetic preparations [10], but this kind of information is vital to understand the role that specialized asexual/clonal reproduction has played in the remarkable success of *A. squamata*. In most allopolyploid parthenogenetic complexes, males appear to be absent even though genetic results allude to occasional sexual reproduction of some kind [4,11,37,59,60,74], but here in this simultaneous hermaphrodite, they are made explicit, raising interesting perspectives regarding the likelihood of reproductive assurance and ploidy change processes such as selfing, gynogenesis and kleptogenesis.

## Data Availability

Table S1 and Figures S1–S6 are in the electronic supplementary material [75]. Data and scripts are located in the Dryad depository [76].
